# Rare presentations of small bowel endometriosis

**DOI:** 10.1002/deo2.395

**Published:** 2024-06-11

**Authors:** Anthony Sakiris, Ezaam Naik Fraz, Arvinf Rajandran, Jason Smith, Benjamin Allen, Sooraj Pillai, Pradeep Kakkadasam Ramaswamy, Sneha John

**Affiliations:** ^1^ Department of Gastroenterology Gold Coast University Hospital Gold Coast Australia

**Keywords:** anemia, abdominal pain, endometriosis, ileal diseases, iron‐deficiency

## Abstract

Despite endometriosis being a relatively common chronic gynecological condition in women of childbearing age, small bowel endometriosis is rare. Presentations can vary from completely asymptomatic to reported symptoms of abdominal pain, bloating, and diarrhea. The following two cases depict very atypical manifestations of ileal endometriosis that presented as obscure intermittent gastrointestinal bleeding and bowel obstruction requiring surgical intervention. The first case describes a previously healthy 40‐year‐old woman with severe symptomatic iron deficiency anemia and intermittent melena. A small bowel enteroscopy diagnosed multiple ulcerated strictures in the distal small bowel as the likely culprit. Despite nonsteroidal anti‐inflammatory drug‐induced enteropathy being initially considered as the likely etiology, histopathological examination of the resected distal ileal segment revealed evidence of endometriosis. The second case describes a 66‐year‐old with a presumptive diagnosis of Crohn's disease who reported a 10‐year history of intermittent perimenstrual abdominal pain, diarrhea, and nausea with vomiting. Following two subsequent episodes of acute bowel obstruction and surgical resection of the patient's stricturing terminal ileal disease, histopathological examination demonstrated active chronic inflammation with endometriosis. Small bowel endometriosis should be considered as an unusual differential diagnosis in women who may present with obscure gastrointestinal bleeding from the small bowel or recurrent bowel obstruction.

## INTRODUCTION

Endometriosis is a common chronic gynecological condition, affecting 10%–15% of women of reproductive age, and is characterized by the histological presence of extrauterine endometrial glands and stroma.^1^ Endometriosis can affect both the large and small bowel. In regard to gastrointestinal endometriosis, the rectosigmoid colon comprises the majority (90%) of cases, while endometriosis involving the ileum is significantly more uncommon (1%–7%).^2^, ^3^ In order to confirm gastrointestinal endometriosis, diagnostic laparoscopy and biopsy are considered the gold standard.^4^ The predominant symptoms of gastrointestinal endometriosis can present widely, and include abdominal pain, bloating, and diarrhea; however, patients can be asymptomatic. The following two cases demonstrate extremely rare presentations of ileal endometriosis that manifested as obscure intermittent gastrointestinal bleeding with associated profound anemia and bowel obstruction requiring surgical intervention. As these presentations can symptomatically overlap with other conditions, such as Crohn's disease, considering small bowel endometriosis as a differential diagnosis is important.

## CASE REPORT

### Case 1

**FIGURE 1:**
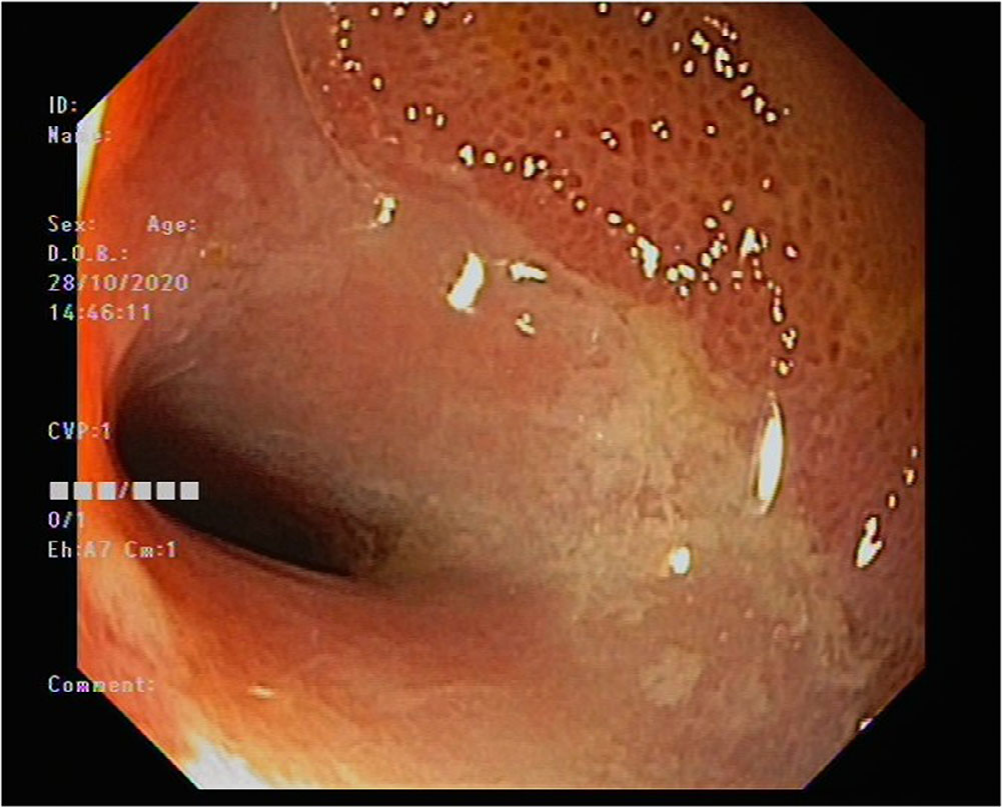
Stricture with circumferential ulceration in the distal ileum.

A 40‐year‐old woman without any significant comorbidities was referred to our department for the investigation of severe iron deficiency anemia. Investigations on presentation demonstrated a hemoglobin of 58g/L; MCV of 67 fL; and ferritin of 2 µg/L. The patient was a non‐vegetarian and denied any gynecological symptoms including menorrhagia. There was no clinical evidence of malabsorption. She reported intermittent episodes of self‐limiting melena with associated fatigue. These symptoms did not correlate with her menstrual cycle. The patient's cross‐sectional imaging, and primary gastroscopy and colonoscopy did not exhibit any relevant pathology. She subsequently proceeded to a capsule endoscopy followed by a retrograde balloon enteroscopy. Multiple ulcerated strictures were detected in a segment of the distal ileum (Figure 1). Histology was non‐specific, demonstrating intestinal mucosa with mild to moderate active inflammation with no architectural atypia or malignancy. Fecal calprotectin levels were occasionally elevated; however, there was no further evidence of inflammatory bowel disease. A provisional diagnosis of nonsteroidal anti‐inflammatory drug (NSAID)‐induced enteropathy was made, as further history exposed regular NSAID use. The patient was managed conservatively with regular iron infusions and ceasing NSAIDs. Despite this, over the next 7 years, she reported intermittent melena with anemia. Additional endoscopic procedures revealed persisting distal ileal strictures. Given these findings and ongoing symptoms, surgery was recommended in order to obtain a definitive diagnosis.

**FIGURE 2:**
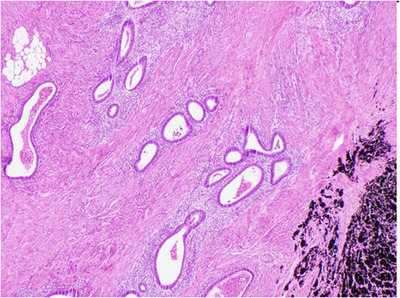
Endometriosis within muscularis propria at the site of stricture and tattoo pigment. Extensive fibrosis, which is often seen in a stricture, is not prominent within this photomicrograph.

Surgical resection was performed and multiple distal small bowel strictures were found. Histopathological examination of the resected bowel demonstrated ileal stricture formation, and endometriosis with endometrial glands and stroma extensively involving the muscularis propria (Figure 2). The mucosal surface showed multiple areas of ulceration and associated inflammation. No Crohn's‐like inflammatory infiltrate or granulomas were identified. There was no evidence of malignancy with 14 negative lymph nodes sampled, and the margins were not involved by endometriosis. Cross‐sectional imaging 2 years following the resection did not exhibit any recurrence of disease.

### Case 2

**FIGURE 3:**
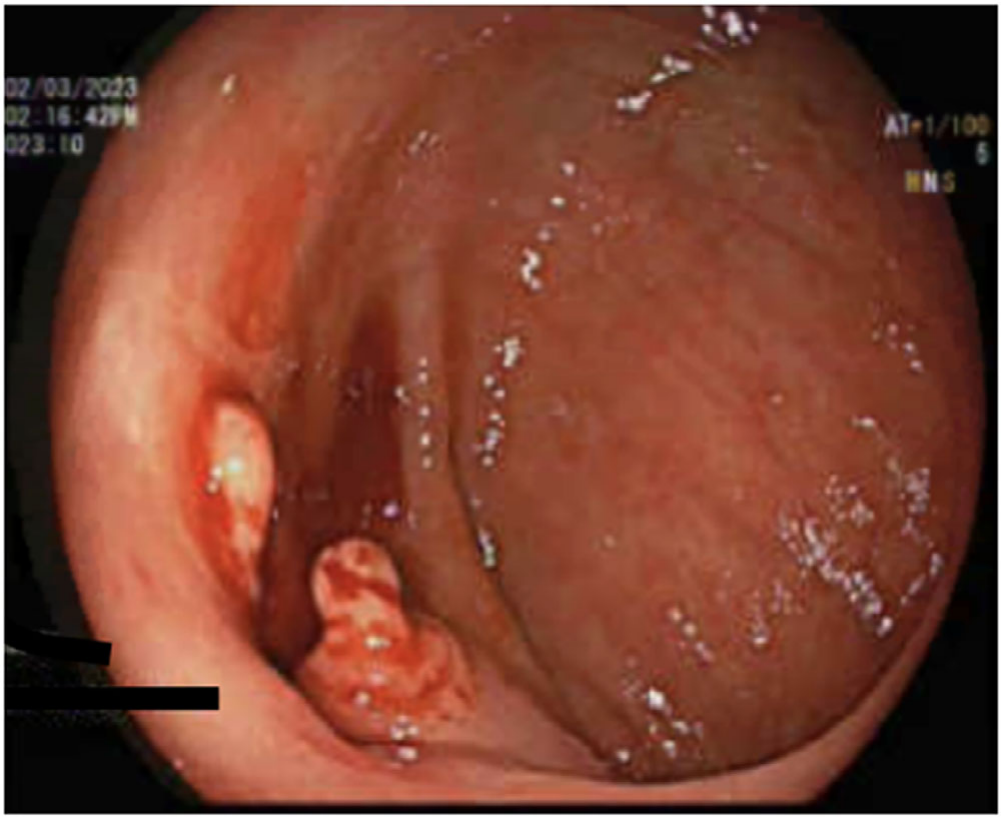
Stricture at the ileocaecal valve.

We report a second case of ileal endometriosis who presented symptomatically with a 10‐year history of intermittent perimenstrual abdominal pain, diarrhea, and nausea with vomiting. A 66‐year‐old woman with psoriasis was referred to our inflammatory bowel disease clinic with a historical diagnosis of presumptive Crohn's disease. Investigations on presentation demonstrated a hemoglobin of 110 g/L, albumin of 29 g/L, CRP of 49 mg/L, and fecal calprotectin of 71 µg/L. Cross‐sectional imaging highlighted nonspecific submucosal fatty infiltration of the terminal ileum. A subsequent colonoscopy revealed a stricture at the ileocaecal valve (Figure 3), with pseudopolyps in the descending colon that were completely removed. Histopathology of the terminal ileum displayed mild active chronic inflammation with an absence of granulomas or dysplasia. Six months after her initial referral, the patient presented with two episodes of acute onset of severe abdominal pain and vomiting due to small bowel obstruction secondary to the ileal stricture. Given the inflammatory component of the stricture, these episodes were successfully managed conservatively with intravenous hydrocortisone. Following the initial bowel obstruction, the patient was commenced on exclusive enteral nutrition and subcutaneous methotrexate.

**FIGURE 4:**
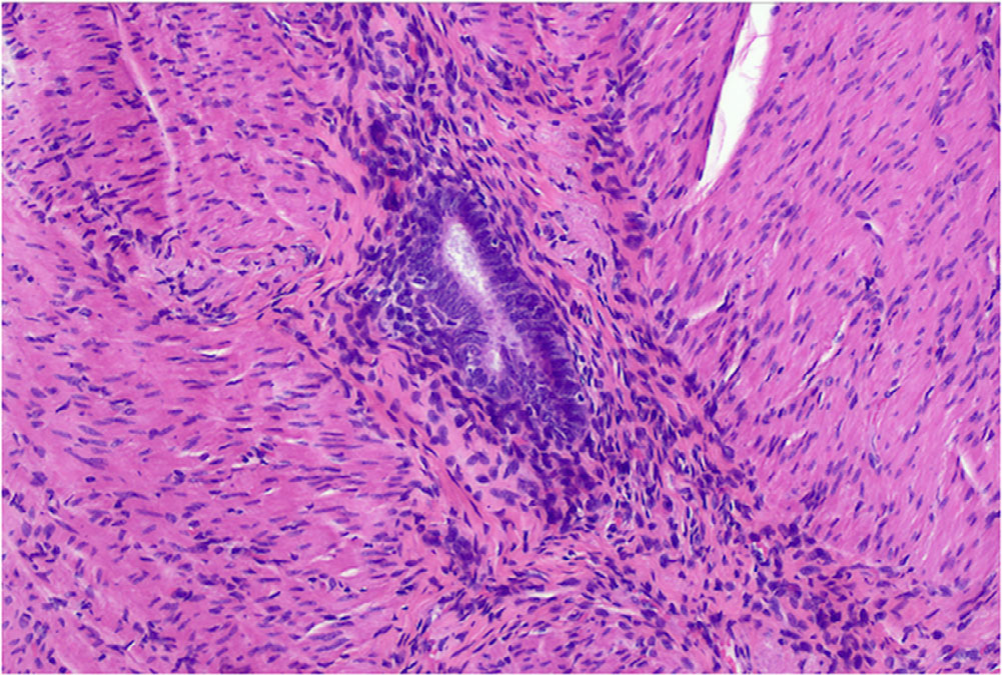
Small bowel endometriosis with endometrial gland and stroma evident (high‐power view, ×200 magnification).

The patient was then referred for an ileocolic resection following a discussion with the Inflammatory Bowel Disease multidisciplinary team.  Operative findings revealed stricturing terminal ileal disease with phlegmon and the cecum adhered to the right lateral pelvic side wall. Histopathological examination of the resected small bowel demonstrated fissuring ulcers and active chronic inflammation. Endometriosis focally involving the terminal ileum (Figure 4) and the appendix was seen. There was no endometriosis involving the mucosa. The surgical resection margins were clear of endometriosis, ulceration, and inflammation. Following surgical resection, surveillance cross‐section imaging had not been performed; however, the patient underwent an intestinal ultrasound that showed normal thickness and motility of the small bowel.

## DISCUSSION

A comprehensive literature review did not describe any cases of small bowel endometriosis as a cause of obscure gastrointestinal bleeding. In addition, only 0.15% of patients with small bowel endometriosis present with bowel obstruction.^5^ Therefore, these two cases are unique and serve as a reminder for clinicians to consider small bowel endometriosis as a differential diagnosis for women who present with unusual recurrent gastrointestinal bleeding or perimenstrual gastrointestinal symptoms including obstructive symptoms. These cases were also atypical due to the patients' endometriosis only affecting a single site with a lack of concurrent gynecological symptoms. In a small study with 93 patients, a single site affecting intestinal endometriosis was only present in 6.5% of patients.^6^ The mechanism of these presentations can be attributed to endometriosis inducing a chronic inflammatory reaction. Inflammatory bowel disease, in particular Crohn's disease, should always remain as a potential diagnosis. NSAID‐induced enteropathy should be considered when there is relevant history of NSAID use. For patients from endemic areas, intestinal tuberculosis should also be considered.

Histological diagnosis of endometriosis obtained from endoscopic biopsies is difficult, as endometriosis extends to the submucosa in 38% of cases and mucosa in only 6% of cases.^7^ For cases of suspected endometriosis wherein the presence of endometrial‐type stroma is uncertain, the utilization of CD10 immunohistochemical staining is a sensitive indicator of endometrial stromal cells at ectopic sites.^8^ Despite this, CD10 staining was not required in these cases for the diagnosis of small bowel endometriosis by the pathologists involved. Recurrence of gastrointestinal endometriosis after surgical resection can vary from 4.7% to 25% over a greater than 2‐year period.^2^ In addition, pregnancy rates following bowel resection can range from 24% to 57%.^2^ Adjuvant treatment following surgical resection to prevent recurrence of endometriosis can include hormonal therapy with danazol or gonadotrophin‐releasing hormone analogs if there are concerns regarding residual disease.^9^ Given the clear margins present in the two cases, adjuvant treatment was not pursued. There is limited consensus regarding surveillance strategies for recurrence of ileal endometriosis following surgical resection; however, monitoring with imaging can be performed if there is a redevelopment of symptoms. Following surgical resection, both patients from these cases have been in clinical remission.

## CONFLICT OF INTEREST STATEMENT

None.

## ETHICS STATEMENT

Written informed consent was obtained from the patients for publication of this case report and any accompanying images.
